# Networks and social capital: a relational approach to primary healthcare reform

**DOI:** 10.1186/1478-4505-5-9

**Published:** 2007-09-25

**Authors:** Catherine Scott, Anne Hofmeyer

**Affiliations:** 1 Knowledge into Action Department, Calgary Health Region, 10101 Southport Road, Calgary, Alberta, T2W 3N2, Canada; 2 Department of Community Health Sciences, Faculty of Medicine, University of Calgary, 3330 Hospital Drive NW Calgary, T2N 4N1, Canada; 3 Faculty of Nursing, University of Alberta 3^rd ^Floor, Clinical Sciences Building, Edmonton, Alberta, T6G 2G3 Canada

## Abstract

Collaboration among health care providers and across systems is proposed as a strategy to improve health care delivery the world over. Over the past two decades, health care providers have been encouraged to work in partnership and build interdisciplinary teams. More recently, the notion of networks has entered this discourse but the lack of consensus and understanding about what is meant by adopting a network approach in health services limits its use. Also crucial to this discussion is the work of distinguishing the nature and extent of the impact of social relationships – generally referred to as social capital. In this paper, we review the rationale for collaboration in health care systems; provide an overview and synthesis of key concepts; dispel some common misconceptions of networks; and apply the theory to an example of primary healthcare network reform in Alberta (Canada). Our central thesis is that a relational approach to systems change, one based on a synthesis of network theory and social capital can provide the fodation for a multi-focal approach to primary healthcare reform. Action strategies are recommended to move from an awareness of 'networks' to fully translating knowledge from existing theory to guide planning and practice innovations. Decision-makers are encouraged to consider a multi-focal approach that effectively incorporates a network and social capital approach in planning and evaluating primary healthcare reform.

## Introduction

Partnerships, collaboratives, interdisciplinary teams, and networks have all been presented as relational strategies to redesign traditional practices, to improve healthcare services and to enhance knowledge exchange between people in healthcare systems [1-8]. The importance of social relationships between people in families, communities, teams, organizations, and other collectives has been well established [9-12] and "identifying the nature and extent of the impact of social relationships is generally referred to as 'social capital' " [10]. For decades, people working in primary healthcare have been encouraged to work in teams, to collaborate with other professionals, to form partnerships with other service providers and other sectors in order to improve health services and health outcomes [13]. More recently, the notion of networks as an important mechanism in and across organizations has made its way into this discourse [14]. As with ideas of collaboration and partnership, there is no consensus within the health services on what is meant by adopting a network approach, and no empirical consensus about the nature of networks [15]. Thus, despite changes in the collaborative rhetoric, little has changed in the way professionals work within and across professional boundaries.

In this paper, we review the rationale for collaboration within healthcare systems; provide an overview and synthesis of key concepts; dispel some common misconceptions of networks; and then finally apply theory to an example of primary healthcare network reform in Alberta (Canada). Our central thesis is that a relational approach to systems change, one based on a synthesis of network theory and social capital can provide the foundation for a multi-focal approach to primary healthcare reform. In the absence of such an approach, collaborative talk alone (e.g., working "in partnership" or "through networks") will do little to enhance practice and bring about real change. It is increasingly apparent that sustained change within health systems requires a multi-focal approach that attends to four elements: (a) motivation of key stakeholders; (b) resources for change; (c) opportunities for change, and; (d) outside motivators [16]. Cohen and colleagues [16] further argue that any attempt to change practice patterns depends upon understanding these four elements and the complex interactions among them. It is our contention that a relational approach to primary healthcare reform encourages health systems decision-makers to think of systems change in terms of reconfiguring and supporting social relations between and among people, groups and organizations. Doing so will establish a foundation for the diffusion of innovative practice patterns that will foster collaborative relationships and improve primary healthcare systems.

## Collaboration in Health Systems

Recognition of the need for collaboration in health systems is not new. Woven throughout discussions of health systems change and knowledge development are references to the role networks play in supporting systems change [2,16,17]. Since the 1970s, health policy shifts have highlighted increasing awareness of the broad determinants of health [18] and health policy documents have emphasized the limits of health systems that work in isolation of other sectors [19,20]. An approach based on the social determinants of health implies working across disciplinary and sectoral boundaries [18]. However, awareness of the need for such an approach and implementation of strategies to engage in such practices have not gone hand in hand. Throughout the 1990s, partnerships were espoused as a way to ensure that health systems were responsive to the social determinants of health [13,18]. It soon became apparent that there was more to working in partnership with other professionals and other sectors, than was initially thought. Barriers to partnership emerged at many levels. The time and resources required and the legal implications (i.e., partners assuming joint liability for the partnership), were but a few of the stumbling blocks encountered. Barriers to collaborative ways of working were often attributed to lack of knowledge and skills at the individual level but it has become increasingly apparent that, even if people have the knowledge, skills, commitment and passion to work collaboratively, these resources will do little where there is limited support within the system for collaborative work [13]. Just as the term partnership became part of common discourse in the 1990s, *networks *and *networking *are terms that are becoming increasingly apparent in public health, primary care, and primary healthcare literature [21-23]. Perhaps part of the attraction to networks as the collaborative relationship of choice is the assumption that networks are informal, naturally occurring relationships that any individual can access. Conversely, establishing formal partnerships with others across conflicted territory is recognized as challenging work.

## Overview and synthesis of key concepts: a networks approach

A network approach is based on an understanding of social structure as patterns of relations (i.e., networks) between social units or actors (i.e., individuals, organizations or countries). The focus of network analysis is on describing social relations and interpreting behaviour based on the relational ties that exist between individuals (i.e., within and between networks) [24,25]. An underlying assumption is that social structures influence the actions of individuals just as the actions can influence social structures. Systematic network analysis therefore helps to describe and explain the constraints and opportunities that social structures impose on individual action [25]. Networks have recently gained attention in the health literature; however, an extensive body of literature has accumulated in other fields over the past thirty years [26]. A large body of literature critiques network theory and the influence of networks on behaviour within the workplace [27,28]; as structures that influence social support [29,30]; and knowledge exchange [2]. Researchers have also studied networks to better understand a variety of risk behaviours in specific populations such as youth at risk [23,24,31]. Within the context of primary healthcare reform, network theory has the potential to guide systems change by drawing on existing theory and incorporating it in decisions about relational change within systems; for example, reconfiguring how professional groups interact and exchange knowledge in the primary healthcare context. Recognition of the potential of network theory will depend on critical application of existing theory and methods.

### Social capital

Szreter and Woolcock said "identifying the nature and extent of the impact of social relationships is generally referred to as 'social capital' ". This view calls into question the "power gradients in society, across which networks may or may not provide links" [10]. Network scholars such as Burt [32,33] describe social capital as the advantage created by the location individuals occupy in the network which is significant in organizations. Social capital refers to resources such as information, support, and social control that flows through networks, rather than the network structure itself [10]. The bonding-bridging distinction was an important development by Gittell and Vidal [34] in the late 1990s to provide a critical lens on social relationships and echoes the earlier distinction made by Granovetter [35] in his theory of the 'strength of weak ties'. The bonding-bridging distinction serves to discriminate between different kinds of social capital and the extent of the impact in terms of cooperation and inclusion [10]. Woolcock [12] provided a useful distinction between:

• Bonding social capital networks have ties that connect people akin to others in similar situations, such as family, friends and neighbours. These close ties provide a sense of identity, affiliation, shared purpose, support and information.

• Bridging social capital networks are about ties that connect people to others who are somewhat distant. These distant ties can span professional boundaries and facilitate access new ideas, information and knowledge.

• Linking social capital networks refers to vertical ties with people who are unlike ourselves and in dissimilar situations, institutions or in positions of authority. These ties allow people to broker useful resources (such as information) across a range of networks and for others to leverage new resources from more distant networks into their existing network [10,36].

One of the key factors determining organizational effectiveness is the nature and extent of the quality of networks between employees and management that influence the employees' ability to access relevant information to effectively solve problems in the workplace [9]. Social capital can lead to new insights when applied to these everyday problems and social challenges.

### Synthesis of key concepts

Essential to the network approach is an understanding that behaviour is embedded in social relationships [2]. Similarly, social capital is about 'social resources or assets' that reside in structures or networks that have measures of cooperation, reciprocity, trust, information and cohesion norms. Of interest to this paper are three key concepts that have emerged from research that illustrate the potential for using network theory to guide knowledge exchange and health systems renewal. This next section provides a synthesis of the following three key networks concepts in the context of social capital, specifically the:(1) strength of weak ties, (2) cross-cutting ties, and (3) structural equivalence or status between individuals who have similar ties with others in a particular network.

#### (1) Strength of weak ties

The notion of the *strength of weak ties *was an early contributor to network theory, and has continued salience. Granovetter [35,37] illustrated that the flow of new information is more likely to come through *weak ties *(i.e., people that are not strongly connected). Granovetter [35] noted that weak ties such as with acquaintances and various contacts were extremely useful in terms of accessing information, opportunities and jobs. Similarly, bridging and linking social capital refer to weaker ties to others in different groups or networks that can provide information and resources to help people in their daily lives [10,11]. Social capital can therefore work as an explanatory factor along with other factors in the context of the problem, (e.g., poor knowledge utilization in health care services). In this discussion, it is also important to consider the implications of a concept this appears antithetical to *weak ties *but which, on closer analysis emphasizes the value of acquaintances and informal contacts. People are less likely to gain new information if they participate in networks that are characterized by redundant connections or *strong interlocking ties *(i.e., people who are strongly connected) and frequent and ongoing contact between limited numbers of similar people who share the same knowledge [37,38]. Granovetter [35] showed that a person's close friends rarely knew more than that person did, so strong network ties served to replicate practice and preserve the status quo. Rogers [7] calls close ties "an interlocking personal network" and notes that "such an ingrown system is an extremely poor net in which to catch new information from one's environment" (p. 309-10). Strong network ties with family and close friends can however, provide more intense support and possibly a greater role in emotional wellbeing [35]. Similarly, bonding social capital refers to strong ties and affliations, but these ties can be a form of social control if the group confers sanctions when individuals do not conform to network norms. Ostracization can limit access to support, information, or other essential resources.

#### (2) Cross cutting ties

Weaker connections between groups represent holes in the social structure. Burt [32] describes structural holes as buffers that insulate networks from one another so that people may remain focused on their specialized tasks. Professional specialization has resulted in health systems that are rife with structural holes. While the ability to focus on areas of specialization may benefit from structural holes, systems that are full of holes may expose people to "differing and inconsistent expectations among multiple constituencies" [28]. In such contexts, it can be difficult to build the cohesion necessary to implement strategic reforms. Burt [32] advocates maximizing the value of structural holes by facilitating opportunities for individuals to build formal, unique ties beyond the group (i.e., to gather new ideas) while implementing strategies to develop cohesiveness within groups (i.e., to generate trust and support).

In health care organizations, natural boundaries exist between specialist medical teams and units, but increasingly, the care of patients with complex morbidities depends on the flow of knowledge across closely bonded networks. Those individuals in boundary spanning roles who operate in the structural holes between teams have 'cross-cutting ties' and credibility with individuals in other networks to broker new ideas and contribute to knowledge exchange. Reforms that require people to adopt new ideas might therefore benefit from tinkering with structures to build bridging ties within or between networks for people immersed in specialized professional activities. It is important to note that it is not just the existence of bridging ties (or brokers) that facilitate the uptake of new information but the quality of those ties. For the past several years, the Canadian Health Services Foundation has been active in its support for the concept of knowledge brokers. The success of funded knowledge broker pilot projects is just beginning to be recognized [39].

#### (3) Structural equivalence/status

Structural equivalence or status in a network reflects the degree to which two individuals have similar relations with others in a network [2,38,40]. People who occupy structurally equivalent positions may not be directly linked with one another but tend to adopt new ideas at a similar level of exposure [2,38]. This is important for administrators and decision-makers because the application of structural equivalence comes into play when planning how to, and who should, introduce new ideas. These are important decisions to achieve buy-in for adopting change. Cognitive determinants such as personal beliefs and team norms influence decisions about adopting new ideas and current research-based evidence. Studies show people prefer to use interpersonal and interactive sources of knowledge such as talking to others who they consider trustworthy, knowledgeable and credible to obtain advice, information or confirm their opinions rather than written sources (which are more often used to disseminate research findings) [41-43]. Deciding what to do is influenced by others who have similar characteristics and who have previously adopted the new knowledge in a successful manner. Quite simply, physicians are more likely to adopt a change in practice (i.e., an innovation) if it has been adopted by other physicians, similarly nurses if it has been adopted by other nurses. This lends theoretical support for the notion that it is crucial to identify the influential champions or peer opinion leaders who are credible with different professional groups to diffuse new ideas. They are trusted, credible individuals who influence the exchange and utilization of research-evidence across networks in organizations. Opinion leaders play a brokering role to accelerate diffusion and transmit information 'by contagion' across boundaries between groups and networks [43,44]. Moreover, individuals with greater interconnectedness across networks typically exhibit greater innovative capacity [7].

These and related concepts provide insight into patterns that exist within networks and draw attention to the influence of structure on behaviour. However, a number of common misconceptions exist that limit the application of a network approach and enable progress from awareness to action. Therefore, we consider six differing perspectives that underpin common misconceptions in the next section.

## Dispel common misconceptions of networks

In order to apply a network approach, we must first critically appraise differing perspectives to move beyond the limited conceptualizations of networks. As an example, we consider six differing perspectives that underpin common misconceptions:

1. The first perspective is that networks are synonymous with organizations [22]. We argue that organizations may encompass many different kinds of ties, such as bonding, bridging and linking ties that exist among individuals and groups with other organizations (e.g., networks that form among administrators, among different professional groups within a clinic, and networks among Region Health Authorities). It is a misnomer to label an organization as a (singular) network. Similarly, networks may form among clinical colleagues for many different reasons (e.g., knowledge exchange through a journal club or communities of practice) but such networks do not necessarily become an organization. When new organizations are formed with the purpose of connecting people and organizations that are addressing similar issues (e.g., the Complementary and Alternative Medicine Education (CAMera) and Research Network of Alberta) they provide the opportunity for people to connect in many different ways (e.g., networks that are formed among providers or users who are interested in particular types of CAM or researchers interested in similar areas of study).

2. The second perspective we challenge is that networks are horizontal, non-hierarchical structures [45]. Social ties take a range of forms, some of which may be hierarchical (e.g., ties formed as a result of reporting structures within organizations or in broader sectors). In some instances it may be possible to pre-determine the structure that ties will take but that is not always the case (e.g., informal short-cuts may be taken within formal reporting structures if it is seen to be more effective, efficient and timely).

3. A third perspective is that networks are based on voluntary participation [46]. In loosely connected networks, individuals may or may not be aware they are part of an extensive network (e.g., an individual may talk with a number of people about administrative decision-making but the members of that network may not all be aware of one another). By way of contrast, participation in a dense network is usually obvious (e.g., ties formed between people who work with one another in a clinical care team on a daily basis). In some instances, membership may be mandated to the point of coercion (e.g., fear of job loss or loss of revenue from a specific source).

4. Another misconception is that networks have decentralized power structures [22,45]. On the contrary, one of the strengths of a network approach is the ability to identify and analyze, not hide, power within networks. Some measures of network centrality are specifically used to determine the relative power of units within a network [25].

5. Another perspective that networks are member controlled and regulated must be challenged [22,45]. The notion of member control is intriguing as it implies a formal structure somewhat akin to the notion of networks as organizations. This level of formality is often not the case and therefore regulation of who is in and who is out is not always feasible.

6. Another perspective is that networks are static so individuals can only be members of one network. But in reality, individuals can be members of many networks such as with family, friends, in their neighborhood, and workplace. In the workplace, it is possible to be a member of more than one network, such as the case of nurse educators who are responsible for two to three patient care units, or nurses who are members of the organization-wide smokers network while also being network members in the patient care unit where they work [47].

These perspectives and assumptions about networks are just a few that permeate the health services literature and limit the transformation from awareness to application (action) of a network approach. In the next section, we apply some of the key network concepts to demonstrate the benefits of a social structural network approach to primary healthcare reform.

## Applying key concepts to primary care networks in Alberta

There is widespread agreement that health system redesign must involve increased emphasis on the provision of health services through primary healthcare models [20,48-51]. In Alberta, as elsewhere, increased emphasis on primary healthcare is based on the understanding that effective models address gaps in health service delivery by coordinating, linking and mobilizing health services to meet the needs of patients [52,53]. A strengthened primary healthcare system is proposed as one mechanism to address limited access to family physicians and fewer medical students choosing to practice family medicine [52,54,55]. Current strategies to strengthen primary healthcare concentrate on the establishment of contractual arrangements between primary care providers, for example between physician groups and Regional Health Authorities, and the implementation of integrated systems for the delivery of primary healthcare services [53,55]. These strategies are evident in Primary Care Networks in Alberta.

### Structure of Primary Care Networks

Primary healthcare reform in Alberta is guided by a groundbreaking 2003 Trilateral Master Agreement among the Alberta Medical Association (AMA), Alberta Health and Wellness (AH&W) and Alberta's nine regional health authorities [49]. The Master Agreement contains four strategic physician agreements, one of which is the Primary Care Initiative Agreement. This agreement between the Regional Health Authorities, the Alberta Medical Association and Alberta Health and Wellness provides incentives for physicians to form alliances and work with the Regional Health Authorities to develop Primary Care Networks [54]. It is proposed that through these Primary Care Networks, comprehensive primary healthcare services will be provided to defined patient populations. Throughout the province of Alberta, over eighteen Primary Care Networks are currently operating or are under development. While the term "network" is being used in planning documents related to this initiative, there is not explicit detail about building capacity in the key concept areas of *strength of weak ties, cross-cutting ties and structural equivalence *(influential peers).

### Physician Alliances

There is much variation in family practice models [56] and it is therefore anticipated that there will be variation in physician alliance models and, by extension, in Primary Care Networks. Physician alliances are formed when a number of practice units develop a loose organizational structure to coordinate and integrate services for their patients as well as people who live in a particular geographic catchment area. Alliances are based on agreements among family physicians that provide similar services for their patient populations. The agreements ensure autonomy of each physician's practice while serving to coordinate and integrate client records, clinical care for unattached patients, urgent care services and to promote the health of the population. As each practice remains autonomous, the creation of governance and communication structures is essential to facilitate decision-making and ongoing working relationships within the alliance and within the region [57]. Weaker ties between networks foster the exchange of knowledge from other areas which improves the information used in the network to serve their patient population. The social capital norms of cooperation, credibility and cohesion further sustain these effective relational ties. Sustainable physician alliances are central to the successful operation of Primary Care Networks [58,59].

Unique practice configurations are determined by history and initial conditions under which the practice was formed, the particular agents involved (i.e., physicians, staff, patients, systems), complex interactions among agents, and regional and global influences [56]. Models for primary care physician alliances and primary care networks have originated primarily in the UK where geography (i.e., size and dispersion of the population) and health system governance differ from the Canadian context [60,61]. A network approach is based on an understanding of patterns of relations between individuals, organizations or countries. While network analysts focus on social structure, they are also interested in how people actively construct relationships that meet their needs [26]. For example, the creation of Primary Care Networks results in the (re)formation of relationships among members of the physician alliances and health region staff so they actively shape structures within which they will work. A physician alliance that is nested within a Primary Care Network provides alternate structures to existing primary care practice models (i.e., sole or group practice). However, little is known about how elements, such as the motivation of key stakeholders, resources for change, opportunities for change, and outside motivators that were outlined by Cohen et al. [16] can be configured to sustain such initiatives within the context of Canadian health care systems [55,60,61]. Identification and description of networks (Table 1) can help to understand these elements of practice change by developing greater understanding of the constraints and opportunities that relational ties impose on individual action [25,26,62].

**Table 1:** Key concepts of a network approach: from awareness toward action in primary healthcare

	**Key concepts**	**From Awareness**	**Toward Action**
**1**	**Strength of weak ties**	• The extent of weak ties between groups are important for leveraging (a) new knowledge from other areas, networks, sectors, and disciplines; and (b) to provide strategies and opportunities for advancing social and career relationships (bridging ties).	• Explicit recognition of the value of weak ties (acquaintances and contacts) as a key to knowledge flow, diffusion and research uptake. • Workplaces can benefit from actively facilitating opportunities for staff to explore a range of contacts in order to address work related issues.

	**Strong interlocking ties**	• Important to consider the extent and nature of close (strong bonding) interlocking ties that may exist within and across the networks. These ties serve to replicate practice, sustain an ingrown system, and preserve structural and procedural status quo norms. • An individual's position in the networks influences their capacity to access resources (e.g., information) to do their job.	• Critically review the impact on practitioners and patient populations when strong bonding ties are more common in primary care networks than weaker ties. • Instigate strategies to mitigate exclusion of those who are sanctioned because they challenge group norms.

**2**	**Cross-cutting ties**	• Weaker connections represent holes in the structures which act as buffers to insulate networks and protect professional specializations. • Health system reform is predicated upon inter-connected teams, knowledge and technology for change, so links through the structural holes are imperative for a better health system. • These diverse ties are important for leveraging resources from powerful individuals and institutions (linking ties).	• While acknowledging that natural ties exist between individuals (e.g., team and disciplinary specialization), it is important to foster cooperative relational ties to diffuse new knowledge. One strategy is to support individuals who have 'weaker connections' that enable them to broker knowledge and influence change in boundary spanning roles across the networks. • It is timely to mentor these individuals and identify what sort of support they require to be effective in the structural holes. • Complexity theory provides useful insights into change and adoption of new practices and research-based knowledge.

**3**	**Structural equivalence/status**	• Ties that exist between individuals with similar characteristics and affiliations. • Important for brokering new ideas and research knowledge/evidence.	• Supporting opinion leaders would be a strategy to introduce new practice and foster support for individuals in an organizational climate of constant change. • Adopting new practice, foster and sustain buy-in across disciplines – teamwork will increase knowledge exchange.

## Conclusion

Despite extensive knowledge and benefits of a network and social capital approach, critical use and analysis of its application remains limited within healthcare systems. Applications remain largely metaphorical, tending to skirt the implications of adopting the network approach. This approach has capacity to make a key contribution to health systems reform and enjoys strong intuitive appeal among decision-makers and health care professionals. But the adoption of an a-theoretical approach will not develop the science and will reduce the heuristic value. Problems arise when advocates create "networks" without fully and critically translating knowledge from existing theory into planning and practice innovations. To (mis)quote Cowen [63] usage of networks based primarily on the warm fuzzy feeling and "glitter [attached to the concept) tends to: (a) break down communication; (b) confuse rather than clarify; and, (c) ultimately retard a field's growth and progress" (p. 3).

In order to avoid such dismal outcomes it is essential that we continue to build a rigorous body of work that extends existing research and demonstrates the significance of a relational approach to systems change based on a synthesis of network theory and social capital. These systems change initiatives would be based on the understanding that primary healthcare structures influence, and are influenced by, the way that people relate to one another. In the Canadian context and beyond, opportunities for change are numerous as health care decision-makers seek approaches to redefine relationships between health regions and among groups of primary healthcare providers [64,65]. Primary health care renewal "requires fundamental changes to the organization and delivery of health care services" and knowledge development and dissemination are key components in system renewal [66]. A clear understanding of the key concepts and benefits of a network and social capital approach provides a promising way to bring together individuals and organizations with a common purpose and goal of primary healthcare reform.

## Competing interests

The author(s) declare that they have no competing interests.

## Authors' contributions

CS was responsible for the paper conception and wrote the first draft. AH collaborated in content and critical revisions to the manuscript. All authors approved the final version of the manuscript.
